# Economics of altering the amount of supplement offered to dairy cows grazing pasture according to nutritional requirements in early lactation

**DOI:** 10.3168/jdsc.2025-0940

**Published:** 2026-03-12

**Authors:** C.K.M. Ho, M.L. Douglas, K. Giri, W.J. Wales, V.M. Russo

**Affiliations:** 1 Agriculture Victoria, Bundoora, VIC 3083, Australia; 2 Agriculture Victoria, Ellinbank, VIC 3821, Australia

## Abstract

This study aimed to determine the effect of grouping grazing-based dairy cows by energy and protein requirements and providing differing amounts of supplements on milk production and income over feed costs. The study was part of a larger experiment that also examined the effect of feeding different starch sources during the first 21 DIM and ensuing early lactation period. The experiment used 108 multiparous spring-calving Holstein-Friesian cows. Cows were grouped into 3 cohorts of equal numbers based on calving date and then randomly allocated to a wheat treatment (n = 18) or a nutritional grouping treatment (n = 90). Cows in the wheat treatment remained in that group throughout the experiment, whereas cows in the nutritional grouping treatment were allocated to 1 of 5 groups after 14 DIM. Cows in the wheat treatment were offered 7 kg DM of wheat grain per day. For cows in the nutritional grouping treatment, milk production and composition, BW, and BCS data from 7 to 14 DIM were used to estimate individual ME and protein requirements. Within each cohort, cows assigned to the nutritional grouping treatment were ranked by nutritional requirements, with the top 12 categorized as high and the bottom 12 as low. Cows were then allocated based on calving date to a high nutritional group (NGH; n = 18) or high control group (ConH; n = 18), or a low nutritional group (NGL; n = 18) or low control group (ConL; n = 18). These 4 groups were compared in the economic analysis conducted in this study. Cows in the nutritional grouping treatment were offered a grain mix in the dairy from 22 to 100 DIM comprising wheat and barley grains and canola meal at 9, 5, or 7 kg DM/cow per day for NGH, NGL, and the controls (ConH and ConL), respectively. Grazed pasture was provided as the balance of the diet with a target allowance of ~25 kg DM/cow per day (measured to ground level). We found no significant effect of grouping by nutritional requirements on milk yield, milk composition, BW, BCS, or change in BW or BCS. There was also no difference in these parameters between the average of the NGH and NGL groups combined and the average of ConH and ConL. However, NGH cows produced more milk than NGL cows (milk yield; 38.6 vs. 35.9 kg/cow per day, ECM yield; 36.8 vs. 34.1 kg/cow per day). We found no effect of the nutritional grouping strategy on profit (milk income minus the cost of grain mix and pasture). However, NGH cows had the numerically highest profit of Australian dollars (AUD) 10.26/cow per day, followed by ConH, NGL, and ConL. The average profitability of the NGH and NGL groups combined, was higher than the average profit of ConH and ConL combined, but the difference was small (AUD 10.19 vs. AUD 10.10/cow per day). Hence, no meaningful economic advantage was determined in this experiment of differentially feeding supplements based on nutritional requirements. Farmers can, therefore, opt for a simpler strategy of providing concentrates at a flat rate to grazing cows without economic penalty during early lactation.

## Highlights

• Grazing dairy cows were fed differing amounts of supplements based on animal demand.
• Cows were grouped by high or low energy and protein needs.
• Cows fed supplements at a differential rate were compared with flat rate feeding.
• Differential feeding showed higher profit, but this was not statistically significant.

## Graphical Abstract

In pasture-based systems, dairy cows are generally offered an amount of concentrate supplements that targets the average cow. This is despite individual cows potentially eating varying amounts and quality of pasture. This study aimed to determine whether offering different amounts of supplement to dairy cows in early lactation according to nutritional requirements can increase farm profit, measured as milk income minus the cost of feed. Cows were categorized into a high or low nutritional group based on their requirements for energy and protein. Our results showed that cows grouped and offered supplements based on their nutritional needs had numerically but not statistically higher profit than cows fed at a uniform rate. DIM = days in milk; DM = dry matter.

**Figure:**
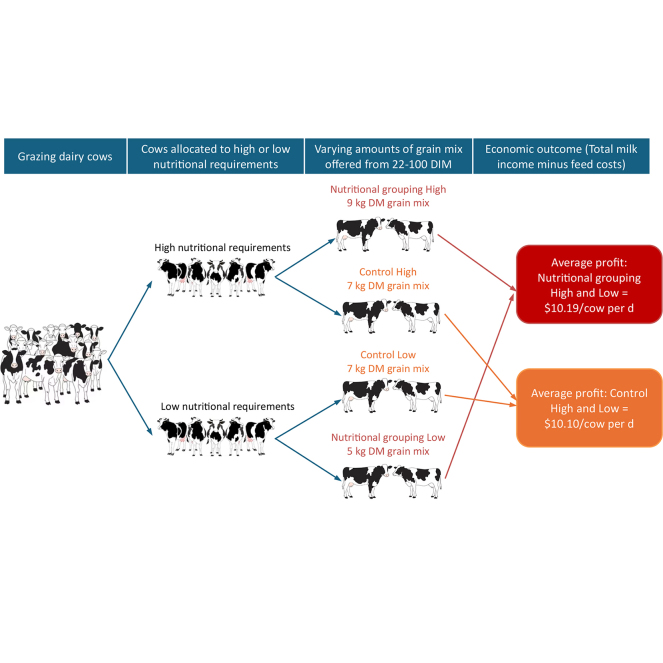

Australian production systems are predominantly grazing-based and are complicated by the fact that individual cows within a herd consume differing amounts and quality of pasture and then receive a fixed amount of supplement, either individually in the dairy or as part of a group on a feed pad (Hills et al., 2015a). The risk of this approach is that all cows are offered an amount of feed that is targeting the average cow, resulting in lower-producing cows depositing additional nutrients as fat and increasing body condition, and higher-producing cows not receiving the nutrients required to meet their milk production potential. Previous research in grazing-based dairy systems has shown that formulated supplement mixes that more closely meet requirements of the herd offer some opportunity to profitably increase production (Douglas et al., 2021; Wright et al., 2024). An alternative strategy is to better match nutrient supply with the nutritional needs of the individual cow. Research from North America has demonstrated that grouping lactating cows according to their nutritional requirements and providing each group with a different TMR can improve milk production and income over feed costs (McGilliard et al., 1983; Cabrera and Kalantari, 2016). This method of grouping, also known as cluster grouping, considers the protein and energy requirements of the cows and has been shown to outperform methods that group by DIM or milk production (Cabrera and Kalantari, 2016). The primary goal of cluster grouping is to minimize the differences in nutrient requirements between cows in the same group while maximizing the differences between groups.

Relatively few studies have compared different supplementary feeding regimens in pasture-based grazing systems (Hills et al., 2015a), and none have considered the economics of offering supplements to grazing dairy cows based on nutritional needs compared with feeding supplements at a flat rate. Recent developments in on-farm technology, animal sensors, and systems to integrate data collection and interpretation could also offer the opportunity to more easily implement differential and individualized feeding approaches (Cabrera et al., 2020). The aim of the study reported here was to determine whether a cluster grouping strategy to categorize cows based on nutritional requirements, can be effectively utilized in a pasture-based feeding system and improve overall herd milk production and farm profit, estimated as milk income minus feed costs in the early lactation period. The hypothesis tested was that farm profit from 22 to 100 DIM will be greater for cows fed tailored amounts of supplements (5 or 9 kg DM/cow per day) according to their nutritional needs compared with cows fed a flat rate of supplement (7 kg DM/cow per day). The experiment was part of a larger study that also investigated the effect on milk production of offering cows starch sources of different fermentability during the first 21 DIM (fresh period) and the subsequent effect during the early lactation period. For completeness, the full experimental design is described herein, but only results relevant to the hypothesis are presented.

The experiment was conducted at the Ellinbank Smartfarm (Victoria, Australia, 38°14′S, 145°56′E). All procedures were conducted in accordance with the Australian Code of Practice for the Care and Use of Animals for Scientific Purposes (National Health and Medical Research Council, 2013). Approval to proceed was obtained from the Department of Energy Environment and Climate Action's Agricultural Research and Extension Animal Ethics Committee (AEC 2021-05). The experiment commenced in July 2021 and used 108 multiparous spring-calving Holstein-Friesian dairy cows. Cows spent the first 4 d postcalving in a colostrum herd before being enrolled in the experiment, where they received ~4 kg DM/cow per day of vetch hay on a feed pad and grazed perennial ryegrass-based pasture. At each milking, cows were offered 2 kg DM of a grain mix via an automated feeding system (De Laval) in the dairy, containing (DM basis) maize (79.9%), canola meal (14.3%), minerals and vitamins (3.7%), magnesium oxide (0.3%), limestone (1.1%), acid buffer (0.6%), and Elitox (0.1%; Impextraco NV, Heist-op-den-Berg, Belgium).

After 5 DIM, cows were allocated into 1 of 3 calving cohorts, with each cohort comprising 36 cows. At this time, cows were also randomly allocated to either a wheat (n = 6 per cohort) or a nutritional grouping treatment (n = 30 per cohort). Cows in the wheat treatment had their maize and canola mix gradually replaced with wheat from 5 DIM until they reached 7 kg DM of wheat only at 9 DIM. They remained on this grain allocation until the completion of the experiment. Cows allocated to the nutritional grouping treatment continued to receive maize and canola meal in the dairy, but at an increased rate of 7 kg DM/cow per day.

For cows allocated to the nutritional grouping treatment, milk production, milk composition, BW, and BCS data from 7 to 14 DIM were used to categorize cows for the early lactation period (22 to 100 DIM) using the cluster method of nutritional grouping as described by McGilliard et al. (1983). Metabolizable energy and protein requirements for each cow were estimated based on CSIRO (2007). Within their cohorts, cows were ranked by nutritional requirements from highest to lowest, with the top 12 categorized as having high nutritional requirements, the middle 6 as medium requirements, and the bottom 12 as low requirements (Figure 1). Cows considered to have either high or low requirements were subsequently allocated, based on calving order, into the high nutritional group (NGH) and high control group (ConH), or the low nutritional group (NGL) and low control group (ConL). The sample size of 18 cows per treatment was calculated to detect a difference in milk yield of 2.4 kg/cow per day, with 72% power at a 5% significance level, based on residual variance from a previous study (Wright et al., 2024).

**Figure 1:**
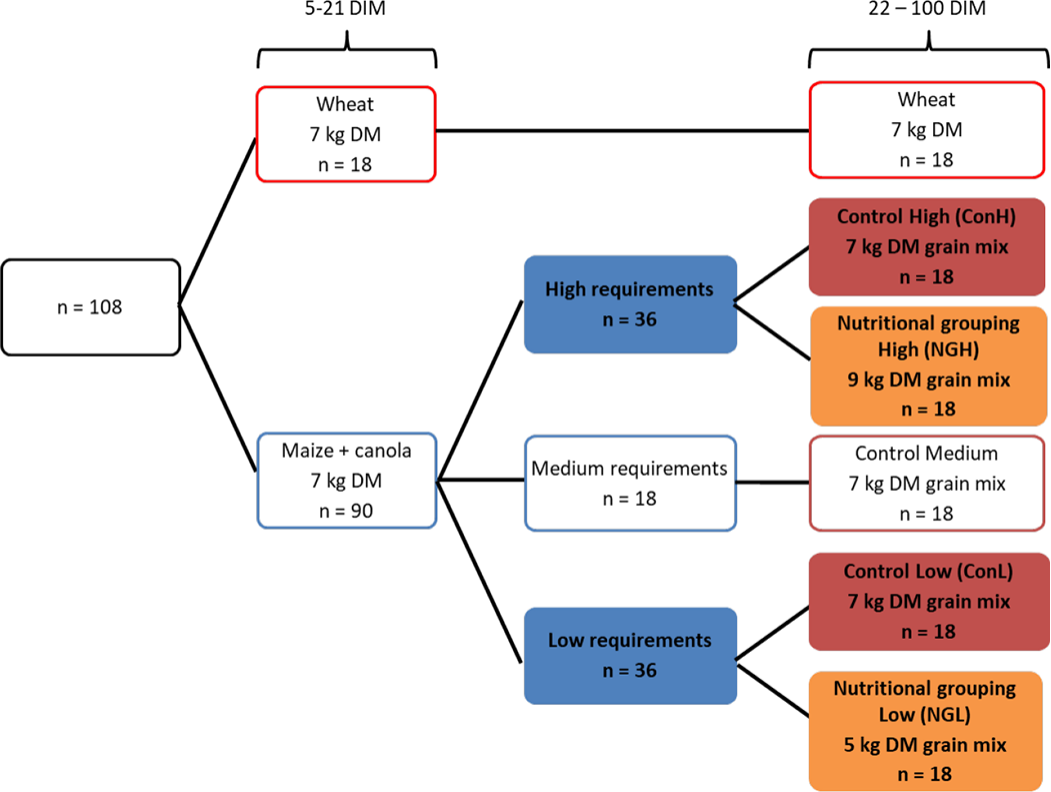
Cow numbers and treatment allocation of the overall experiment, with shaded boxes indicating nutritional grouping treatments.

Cows assigned to the nutritional grouping treatment were offered a grain mix in the dairy from 22 to 100 DIM that comprised (DM basis) wheat (23.1%), barley (46.2%), canola meal (23.1%), minerals and vitamins (3.4%), magnesium oxide (0.3%), limestone (1.0%), canola oil (0.6%), and Elitox (0.1%). The grain mix was offered at 9 kg DM/cow per day for NGH cows, 5 kg DM/cow per day for NGL cows, and 7 kg DM/cow per day for ConH and ConL cows. The balance of the diet was offered as grazed pasture, with each cohort grazing as an individual herd. Cows were offered a pasture allowance of ~25 kg DM/cow per day, measured to ground level. Daily allowances were divided into equal a.m. and p.m. grazing breaks and were fenced to prevent back-grazing. Each cow was changed from the fresh cow treatment to the early lactation treatment on an individual basis (i.e., as the individual cow reached 22 DIM).

Individual cow DMI of pasture was estimated during a 5-d measurement period using the n-alkane technique (Mayes et al., 1986). The measurement period was staggered between the cohorts with a mean DIM of 49 ± 3.9 (mean ± SD) at the beginning of the measurement period. Each cow was dosed with a known amount of a synthetic n-alkane (C_32_) twice daily for 10 d, which included the measurement period as well as the 5 d prior. Samples of the pasture, grain mix, and feces were collected twice daily for the 5-d measurement period. Pasture samples were collected by cutting multiple subsamples of pasture offered to estimated grazing height per day. Grain offered was collected at each milking for each cohort. Samples of grain mixes and pasture were stored immediately following collection at −18°C and subsequently freeze-dried at −55°C for a minimum of 72 h. Following freeze drying, samples of pasture and grain mix offered were ground through a 1-mm screen. Fecal samples were stored at −18°C, and after completing collection, samples were defrosted before being oven-dried for 72 h at 40°C. Once dried, samples were ground through a 1-mm screen then bulked into a 10-g sample per cow and analyzed for n-alkane concentrations.

Concentrations of n-alkanes were analyzed by GC using the methods described by Dove and Mayes (2006) and Liu et al. (2022). Peak areas were converted to amounts (mg/kg DM) of n-alkane by reference to the internal standard (tetratriacontane, C_34_). Individual pasture intake was estimated using the following equation:

$$\mathrm{intake}\;(\mathrm{kg}\;\mathrm{DM}/\mathrm{cow}\;\mathrm{per}\;\mathrm{day})=\frac{\left(\frac{F_{i}}{F_{j}}\times \left(D_{j}+I\times G_{j}\right)-I\times G_{i}\right)}{\left(P_{i}\;-\frac{F_{i}}{F_{j}}\times P_{j}\right)},$$

where F_i_, P_i_, and G_i_ are the fecal, pasture, and grain mix concentrations of odd-chain n-alkane (C_33_), respectively; F_j_, P_j_, and G_j_ are the fecal, pasture, and grain mix concentrations of even-chain length alkane C_32_, respectively; and D_j_ is the daily dose of even-chain C_32_ alkane. Grain mix intakes (I) were entered.

The amount of grain mix refused was measured at each milking for each cow during the experiment period. Individual DMI of the supplement was calculated as the difference between the amounts of grain mix offered and refused. Representative samples of grain offered were collected daily (separate samples for each cohort) during the intensive measurement period and fortnightly outside of this period to determine DM percentage and nutrient concentrations. Samples collected for determining DM percentage were weighed and placed in an oven at 105°C for 48 h, then weighed again upon removal. Samples for nutrient concentrations were stored at −18°C. Grain samples were then oven-dried at 60°C until dry and ground through a 1-mm sieve and sent to a commercial laboratory (Dairy One Forage Laboratory, Ithaca, NY, via Feed Central) for nutrient analysis via wet chemistry (Dairy One, 2022).

During the intensive measurement period, pregrazing pasture mass was 4,270 kg DM/ha, postgrazing pasture mass was 1,590 kg DM/ha, and daily herbage allowance was 27 kg DM of pasture per cow, measured to ground level. During the extensive period, pregrazing pasture mass was 3,780 kg DM/ha, postgrazing pasture mass was 1,760 kg DM/ha, and daily herbage allowance was 29 kg DM of pasture per cow. Pre- and postgrazing pasture mass was determined by rising plate meter (**RPM**; Earle and McGowan, 1979). During the intensive measurement period, pre- and postgrazing pasture mass were estimated once per day by taking ~150 readings per paddock using an RPM in a zigzag pattern across the paddock. Outside of this period, estimates of pre- and postgrazing pasture mass were made once every 2 wk in the same way. Quadrant cuts were taken in each new paddock during the intensive measurement period and every 2 wk during the extensive period to construct calibration equations by plotting pasture mass against RPM readings.

Samples for the assessment of offered pasture nutrient concentrations were collected using battery-operated hand shears to cut spot samples (toe-cuts) at a transect across the paddock (~30 samples per paddock). Pasture samples were cut at estimated grazing height (5 cm above ground level) and were taken daily during the intensive measurement period and fortnightly outside of this period. The samples were washed and stored at −20°C before being oven-dried at 60°C until dry, then ground through a 1-mm sieve for nutritive analyses. Analyses were conducted via wet chemistry at a commercial laboratory (Dairy One Forage Laboratory, Ithaca, NY, via Feed Central).

Milk yield of each cow was recorded twice daily at ~0600h and 1500h using a DeLaval milk metering system (MM27BC, DeLaval International, Tumba, Sweden). Samples of milk (p.m. and a.m.) were collected using in-line milk meters (DeLaval International, Tumba, Sweden) twice weekly (4 milkings). Milk samples were sent to a commercial laboratory (HICO, Korumburra, Australia) to be analyzed for fat, protein, and lactose using a mid-infrared milk analyzer (Bentley FTS, Bentley Instruments, Chaska, MN). Energy-corrected milk, standardized to 4.0% fat and 3.3% protein, was calculated using the following formula (Tyrrell and Reid, 1965):

ECM (kg/cow per day) = milk yield kg × (376 × fat% + 209 × protein% + 948)/3,138.

Walkover scales (DeLaval Automatic Weight System AWS100, Tumba, Sweden) were used to measure cow BW, and an automatic 3-dimensional camera (DeLaval Body Condition Scoring, BCS DeLaval International AB, Tumba, Sweden) was used to measure BCS. Both measurements occurred daily following the morning milking. Raw data from the BCS camera were refined by fitting a robust smooth loess function to identify and remove outliers as described in Albornoz et al. (2022). The 8-point BCS scale of Earle (1976) was used.

In a similar approach to Ho et al. (2018), the economic analysis assumed the grain mixes would be fed via existing infrastructure present in the dairy, and the only additional costs were for the supplement and pasture. The profit indicator used was total income from milk produced minus the cost of grain mix plus pasture and was calculated daily for each cow. Milk income comprised the sum of the separate contributions from milk protein and milk fat as measured in the experiment. To calculate feed costs, the amount of grain mix offered to cows in the experiment was used, whereas the amount of pasture was estimated using the back calculation method (Heard et al., 2011), where

$$\begin{array}{c} \mathrm{pasture}\;\mathrm{DMI}\;(\mathrm{kg})\;=\;\\ \frac{\left(\mathrm{livestock}\;\mathrm{ME}\;\mathrm{requirements}\;-\mathrm{ME}\;\mathrm{from}\;\mathrm{supplementary}\;\mathrm{feeds}\right)}{\mathrm{ME}\;\mathrm{content}\;\mathrm{of}\;\mathrm{pasture}}. \end{array}$$

Livestock ME requirements comprised those for maintenance, activity, lactation, growth, and body tissue change, and were estimated using CSIRO (2007). The ME requirements for pregnancy are minimal in the early lactation period and therefore ignored. Weekly pasture DMI was estimated for each cow using the milk production, BW, age, BCS change, and grain mix intake data measured during the experiment. Terrain was considered as undulating and the average distance walked by the cows per day was 4 km. The ME concentrations of grain mixes and pasture were obtained from the respective chemical analysis undertaken.

The income from milk produced and cost of feed was expressed in Australian dollars (**AUD**; average exchange rate at the time of analysis: AUD 1 = USD 0.75). Milk prices and supplementary feed costs were based on historical data from Dairy Australia (2020, 2021), with input from a group of industry experts comprising farmers, service providers, scientists and economists. The milk price used was AUD 6.37/kg protein + fat (AUD 9.09/kg protein and AUD 4.13/kg fat) and represents the average of typical prices paid in Victoria between 2010 and 2020, after adjusting for inflation (Reserve Bank of Australia 2020). Feed prices for wheat grain (AUD 370/t DM), barley grain (AUD 343/t DM), and canola meal (AUD 496/t DM) were based on the mean of distributions derived from weekly prices between 2015 and 2020, converted to 2020 dollars. Distributions were fitted using @Risk, an add-in package to Microsoft Excel (Palisade, 2018). Pasture is an intermediate input within the farm system rather than a directly traded output, and as such, can be difficult to value (Lewis et al., 2020). In the absence of historical data to develop a price distribution, the panel of industry experts estimated an average price of AUD 150/t DM for pasture, drawing on market values of close substitutes. The treatments compared in the economic analysis were ConH, NGH, ConL, and NGL. In addition, NGH and NGL were considered in combination relative to ConH and ConL combined, to provide an indication of the profitability of grouping and feeding supplements to cows based on nutritional needs compared with offering cows the same amount.

Before statistical analyses, daily data on milk production, BCS, BW, and profit were averaged over the early lactation period providing a mean value for each cow. Both these mean data per cow and the individual average pasture DMI measured by the n-alkane technique were analyzed by analysis of covariance (**ANCOVA**) model in Genstat 22 (VSN International Ltd., Hemel Hempstead, UK). These ANCOVA models had treatment as main effect and cohort and cow within cohort as blocking structure. The effect of the same variable measured in the covariate period (average per cow in covariate period), if available, was fitted as a covariate to improve the precision of the estimates. Specific contrasts for the combination of treatment means were formed and used to test specific hypotheses concerning nutritional groupings. The statistically significant difference between 2 treatments were presented by different letters based on Fisher's unprotected LSD test at 5% level of significance. The histograms of residuals and plots of residuals versus fitted values were examined for normality of residual distribution with constant variance.

Dry matter intake, milk production, BW, and BCS for the nutritional grouping treatments as compared in the economic analysis are given in Table 1.

**Table 1:** Mean daily yield of milk and ECM, concentrations and yields of milk fat and protein, DMI, BW, δBW, BCS, δBCS, and profit measured over the early lactation period (22–100 DIM)

Item	Treatment				SED^1^	*P*-value
	Control high	Nutritional grouping high	Control low	Nutritional grouping low		
Grain mix DMI (kg DM/cow per day)	7.0^c^	8.7^d^	7.0^c^	5.3^a^	0.078	<0.001
Pasture DMI^2^ (kg DM/cow per day)	17.4^b^	16.2^ab^	17.3^ab^	17.3^ab^	0.756	0.119
Total DMI^2^ (kg DM/cow per day)	24.4^b^	24.9^b^	24.3^b^	22.6^a^	0.750	0.001
Milk yield (kg/cow per day)	38.6^bc^	38.9^c^	37.8abc	35.9^a^	1.236	0.106
ECM yield (kg/cow per day)	37.0^bc^	36.8^c^	35.5abc	34.1^ab^	1.109	0.040
Milk fat yield (kg/cow per day)	1.37^b^	1.41^b^	1.36^b^	1.32^ab^	0.050	0.018
Milk protein yield (kg/cow per day)	1.19^ab^	1.22^b^	1.17^ab^	1.13^a^	0.036	0.159
BW (kg)	581^a^	580^a^	585^a^	575^a^	7.357	0.853
δBW^3^ (kg)	−24.1^a^	−25.2^a^	−19.8^a^	−29.7^a^	7.360	0.853
BCS	4.31^a^	4.37^a^	4.37^a^	4.37^a^	0.036	0.495
δBCS^3^	−0.25^a^	−0.19^a^	−0.19^a^	−0.19^a^	0.036	0.495
Profit^4^ (AUD/cow per day)	10.16^a^	10.26^a^	10.05^a^	10.12^a^	0.376	0.856

a–d Means within a row with different superscripts differ significantly based on Fisher's unprotected LSD test at 5% level of significance.

1 SED = standard error of the difference.

2 Measured over a 5-d measurement period only.

3 Difference between means of the early lactation and covariate periods.

4 Profit = total milk income minus cost of grain mix and pasture.

By design, the intake of grain mix was higher for cows on NGH and lower for cows on NGL than the intake of grain mix for cows on either ConH or ConL (Table 1). Based on contrast testing, pasture DMI of cows measured during the intensive measurement period was not different between cows fed varying amounts of supplements based on nutritional needs (NGH and NGL) and the ConH and ConL cows that were fed at a flat rate (*P* = 0.252), nor was there any difference when comparing NGH with ConH or NGL to ConL (*P* = 0.106 and *P* = 0.998, respectively) for pasture DMI when tested as contrasts. However, pasture DMI was numerically lowest for cows on NGH. Due to the difference in grain offered (7 kg DM vs. 5 kg DM/cow per day) total DMI was greater for ConL cows compared with NGL cows (*P* = 0.011, 24.3 vs. 22.3 kg DM). Total DMI was not different for NGH, ConH, and ConL.

Mean yields of milk, ECM, and milk composition are presented in Table 1. Observed milk production over the experimental period is presented in Figure 2. We found no significant effect of the nutritional grouping strategy on milk yield, ECM yield, milk composition, BW, BCS, or change in BW or BCS. We also found no difference in these parameters between the average of NGH and NGL combined and the average of ConH and ConL. However, cows on the NGH treatment produced more milk than cows on NGL. No differences were observed between any other treatments.

**Figure 2:**
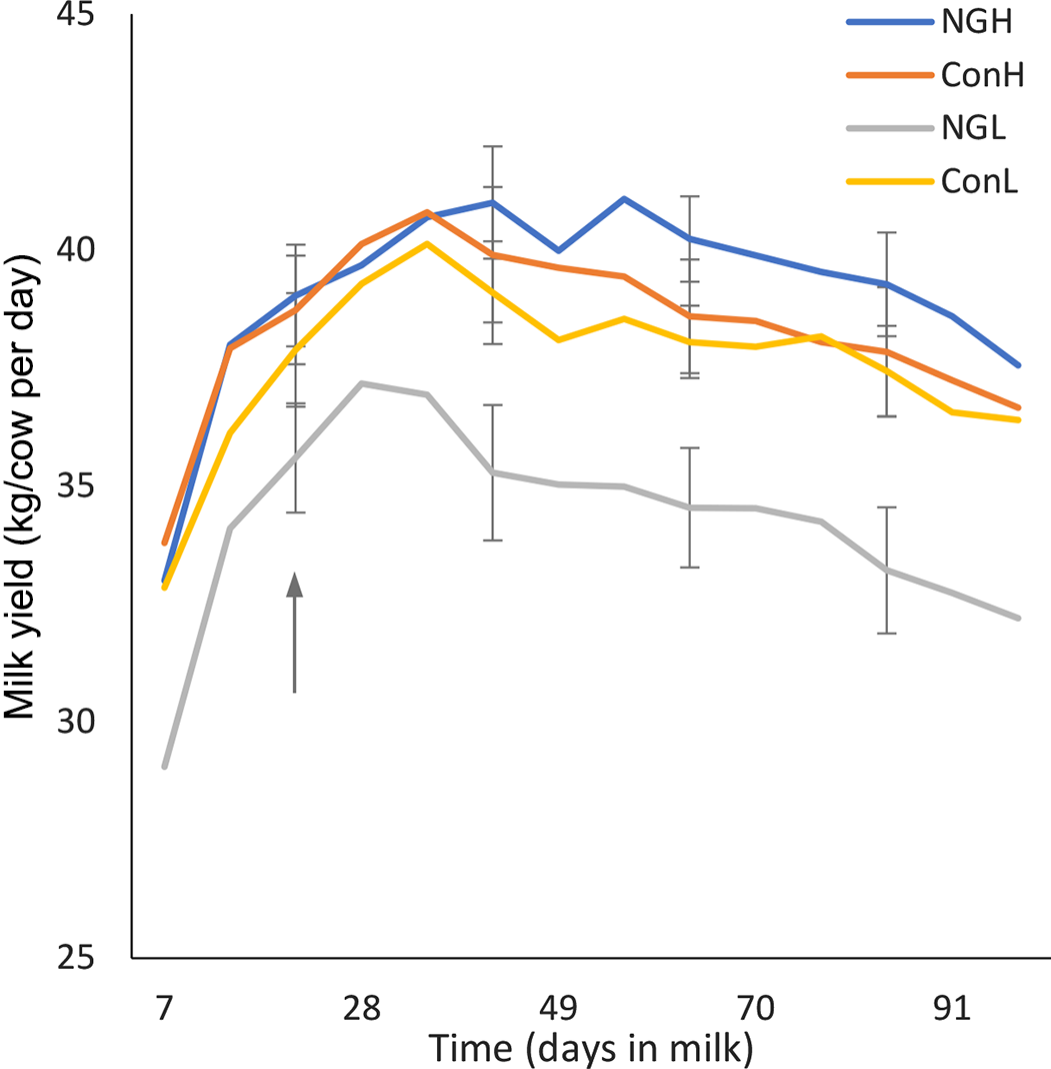
Observed milk production over the experimental period (5–100 DIM) for cows in the high nutritional group (NGH), high control group (ConH), the low control group (ConL), and low nutritional group (NGL). Arrow indicates when early lactation treatments commenced. Error bars indicate the SEM on the relative day.

Mean daily profit of the treatments during the early lactation period is given in Table 1. We found no effect of the nutritional grouping strategy on profit. Cows on the NGH treatment had the numerically highest profit of AUD 10.26/cow per day, followed by ConH, NGL, and ConL. Although the average profitability of cows in the NGH and NGL groups combined was higher than the average profit of cows in the ConH and ConL groups combined, the difference was small (AUD 10.19/cow per day vs. AUD 10.10/cow per day).

Previous studies have demonstrated variability in profit among cows and suggested that this provides the opportunity to differentially feed individual animals (McGilliard et al., 1983; André et al., 2010). The ability to take advantage of these differences has been identified as a critical determinant for the successful application of differential feeding (Hills et al., 2015b). Positive milk production and economic benefits from feeding cows based on nutritional requirements have been shown in TMR systems (Maltz et al., 2013; Kalantari et al., 2016), but the results of similar studies in pasture-based systems have been varied. For example, Dale et al. (2016) and Dela Rue et al. (2025) found no improvement in milk production, but García et al. (2007) measured an increase in milk yield and milk solids production. In the experiment reported here, cows separated into high and low nutritional requirement groups and fed different amounts of supplements during the early lactation period had numerically but not statistically higher profit than cows fed a flat rate of concentrate, and therefore the hypothesis tested is rejected.

A possible contributing factor to the lack of economic benefit observed from the nutritional grouping strategy could be the relatively generous pasture allowance offered to cows in the experiment. Although the pasture allowances offered were within the range typically recommended for perennial ryegrass in Australia and New Zealand (DairyNZ, 2023), cows offered the NGH treatment had numerically lower pasture DMI than cows in the ConH group, demonstrating some potential substitution of pasture for supplement. Stockdale (2000) showed that substitution increased by 0.16 kg DM for each kilogram of concentrates. It has previously been argued that a restricted pasture allowance to reduce the effect of substitution may be needed to fully realize the response to supplements, and therefore the benefits of differential feeding (Hills et al., 2015b). Restricted pasture allowance creates competition between cows, such that cows requiring higher nutritional requirements will be most affected and offering additional supplements should improve milk production as a result of better meeting nutritional demand. Correspondingly, for cows with lower nutritional requirements, the substitution of pasture for supplements would be reduced with a lower amount of supplement offered. Future investigations of the nutritional grouping approach in pasture-based systems could involve other times of the year when pasture quantity and nutrient concentrations are more limiting (e.g., autumn), where the response to supplements is likely to be more pronounced.

In this experiment, animals were allocated to their nutritional groups based on cow data measured during the fresh period, a time when the cow experiences a range of metabolic, physiological, management, and environmental challenges as she changes from a pregnant nonlactating animal to a high-producing dairy cow (Albornoz et al., 2024). Therefore, the nutritional requirements at this time may not reflect those later in lactation. Cows also remained in their assigned treatment group for the duration of the experiment. Adjusting the nutritional groupings before cows reached 100 DIM, or after this, could have provided some benefit. In their review of the value of nutritional grouping for TMR systems, Cabrera and Kalantari (2016) noted that the frequency of grouping events has varied across studies from once during a lactation, to every few months, to monthly or weekly. However, no studies have examined the effect of timing and frequency of grouping events on animal performance and the subsequent economic impact. The extent to which this is critical in the application of nutritional grouping approaches within pasture-based systems warrants further investigation.

A partial budget approach was used to conduct the economic analysis in this study, where it was assumed the necessary capital to feed cows variable amounts of supplement in the dairy was already in place. This approach is consistent with other analyses examining the profitability of nutritional grouping strategies (e.g., Kalantari et al., 2016; Wu et al., 2019). However, for farmers planning to invest in an individualized feeding system, a more complete evaluation that estimated the net present value and internal rate of return of such an investment could be worthwhile (Heard et al., 2012; Ferreira et al., 2016). These metrics would account for the costs of the infrastructure and equipment, the time value of money, the opportunity cost of investing that capital elsewhere, as well as any additional benefits of the system at other times of the year. In seasonal pasture-based dairy systems, investments that enhance BCS or improve reproductive outcomes can be particularly valuable, as reproductive efficiency underpins compact calving and efficient pasture utilization, as well as contributes to the overall productivity and profitability of the farm business (Butler et al., 2019; Ruelle et al., 2021). These additional benefits can be important considerations, as farmers are often budget-constrained and seeking to use the available resources in their businesses in the most efficient way for the longer term (Malcolm, 2004).

In summary, this study compared grazing-based dairy cows that received supplements adjusted to their nutritional needs with cows fed a fixed amount of concentrate. Although cows classified as having high or low nutritional needs showed slightly higher average profitability than those on a flat rate feeding strategy, the difference was not significant, indicating no clear advantage to differential supplementation in this experiment. Therefore, farmers can adopt a simpler approach of flat rate feeding of concentrates to grazing cows without a significant economic penalty during the early lactation period.
